# Autonomous exercise game use improves metabolic control and quality of life in type 2 diabetes patients - a randomized controlled trial

**DOI:** 10.1186/1472-6823-13-57

**Published:** 2013-12-10

**Authors:** Kerstin Kempf, Stephan Martin

**Affiliations:** 1 West-German Centre of Diabetes and Health, Düsseldorf Catholic Hospital Group, Hohensandweg 37, 40591 Düsseldorf, Germany

**Keywords:** Type 2 diabetes mellitus, Overweight, Obesity, Exercise game, Lifestyle intervention, Non-pharmacological therapy, HbA1c, Weight loss, Quality of life

## Abstract

**Background:**

Lifestyle intervention in type 2 diabetes mellitus (T2DM) is effective but needs a special local setting and is costly. Therefore, in a randomized-controlled trial we tested the hypothesis that the autonomous use of the interactive exercise game *Wii Fit Plus* over a period of 12 weeks improves metabolic control, with HbA1c reduction as the primary outcome, and weight loss, reduction of cardiometabolic risk factors, physical activity and quality of life (secondary outcomes) in T2DM patients.

**Methods:**

Participants (n = 220) were randomized into an intervention and a control group. The intervention group was provided with a *Wii* console, a balance board and the exercise game *Wii Fit Plus* for 12 weeks. The control group remained under routine care and received the items 12 weeks later. At baseline and after 12 weeks (and for the control group additionally after 12 weeks of intervention) the participants’ health parameters, medication, physical activity and validated questionnaires for quality of life (PAID, SF12, WHO-5, CES-D) were requested and compared in a complete case analysis using the Mann–Whitney test and the Wilcoxon signed rank test.

**Results:**

80% of participants completed the 12-week study. Patients in the intervention group significantly improved HbA1c (from 7.1 ± 1.3% to 6.8 ± 0.9%; -0.3 ± 1.1%; p = 0.0002) in comparison to the control group (from 6.8 ± 0.9% to 6.7 ± 0.7%; -0.1 ± 0.5%) and also significantly reduced fasting blood glucose (from 135.8 ± 38.9 mg/dl to 126.6 ± 36.6 mg/dl; p = 0.04), weight (from 97.6 ± 19.2 kg to 96.3 ± 18.7 kg; p < 0.001) and body mass index (from 34.1 ± 6.5 kg/m^2^ to 33.5 ± 6.5 kg/m^2^; p < 0.001). Daily physical activity increased significantly (p < 0.001). Diabetes-dependent impairment, mental health, subjective wellbeing and quality of life also improved significantly, and the number of patients with depression decreased. Similar improvements were seen in the control group after exercise game intervention.

**Conclusions:**

In this trial a low-threshold intervention with the interactive exercise game *Wii Fit Plus* was able to motivate T2DM patients to improve physical activity, glucometabolic control and quality of life.

**Trial registration:**

ClinicalTrials.gov NCT01735643.

## Background

Type 2 diabetes mellitus (T2DM) can be successfully treated by lifestyle change [1]. However, comprehensive lifestyle interventions need local settings and are cost intensive. Instead of early and increasing anti-diabetic medication, motivational, low-threshold interventions are needed. Interactive exercise games (exergames) are a new and alternative tool to promote physical activity. They can be interfaced with a television set and offer different games (e.g. table tennis, bowling) in order to substitute sedentary leisure time with active time. There are some reports addressing health benefits of exercise gaming [2], however, data are lacking for clinical endpoints of diseases (i.e. HbA1c) or possible effects for quality of life. Elderly T2DM patients are particularly interesting candidates for exercise games because of their usually sedentary lifestyle [3]. Therefore, in a randomized-controlled trial we investigated the hypothesis that autonomous use of the interactive exercise game *Wii Fit Plus* over a period of 12 weeks is able to improve HbA1c (primary outcome) as well as weight, cardiometabolic risk factors, physical activity and quality of life (secondary outcomes) in T2DM patients.

## Methods

### Study subjects

T2DM patients were recruited throughout Germany via attending physicians or newspaper articles. Eligible patients (inclusion criteria: diabetes duration <5 years, aged 50–75 years, BMI ≥ 27 kg/m^2^, subscribed in the disease management program (DMP) T2DM; exclusion criteria: regular physical activity, pharmacological diabetes therapy with the exception of Metformin or DPP-4 inhibitors) were randomized according to an electronically generated randomization list (created by trial statistician) into parallel groups (assigned by study nurse). In detail, each participant was assigned a serial study ID. For each ID there was a closed envelope with the group assignment. Both the participants and the study nurse were blinded for sequence of allocation concealment. The intervention group was provided with a *Wii* console, a balance board and the exercise game *Wii Fit Plus* (Nintendo of Europe GmbH, Frankfurt am Main, Germany) with the instruction to use these items for at least 30 min/day during the next 12 weeks. The control group remained under routine care (i.e. quarterly visits to the attending physician according to the DMP T2DM with check of HbA1c, weight, BMI, blood pressure, and blood lipids and therapy adjustment if necessary) and received the intervention 12 weeks later. At baseline and after intervention (and for a secondary analysis additionally for the control group: after 12 weeks of waiting phase) health parameters (i.e. weight, BMI, and blood pressure) were measured by the attending DMP physician. Body weight was measured in light clothing to the closest 0.1 kg, and height to the closest 0.5 cm. Blood pressure was measured after a five-minute rest in a sitting position on both arms. Venous blood was collected after an overnight fast of 8 h by inserting an intravenous cannula into the forearm vein, and laboratory parameters (i.e. HbA1c, levels of fasting blood glucose (FBG), triglycerides, total-, HDL- and LDL cholesterol) were analyzed at local laboratories. Self-reported physical activity was assessed according to a 6-point questionnaire asking for intensity of physical activity during working and leisure time, frequency of physical activity in summer and winter, duration of walking and bicycling time per day during the last 12 weeks. Single values were summed up with the maximum reachable value set to 100%. Participants in both groups filled out validated self-assessment questionnaires for diabetes-dependent impairment (Problem Areas in Diabetes (PAID) [4], physical and mental health (SF-12) [5]. subjective wellbeing (WHO-5) [6], and quality of life (German version ADS-L (Allgemeine Depressionsskala) of the general depression scale CES-D [7]). Depression was defined to be present at a score >39% (PAID), ≤52% (WHO-5) and ≥40 (ADS-L). Primary endpoint was the within-group difference of HbA1c after 12 weeks of intervention compared between the exercise game intervention group and the waiting control group; secondary endpoints were differences in weight, cardiometabolic risk factors, physical activity and quality of life measurements. Results from the waiting controls after their 12 weeks of exercise game intervention were analyzed separately in a secondary analysis. The first participant was enrolled on 14.01.2011; the last subject finished the intervention on 18.09.2012. The research protocol was approved by the ethics committee of the Ärztekammer Nordrhein, carried out in Düsseldorf in accordance with the Declaration of Helsinki and all participants gave written informed consent.

### Statistical analysis

Sample size had been determined by Simple-Interactive-Statistical-Analysis (http://www.quantitativeskills.com/sisa/calculations/samsize.htm) assuming that T2DM patients can improve their HbA1c by using the exercise game *Wii Fit Plus*. Our own data indicated that by motivation for lifestyle change a reduction of 0.7% ± 1.2% of HbA1c could be achieved [8], while for the control group a reduction of only 0.2% was assumed. To be able to measure such a reduction with a power of 90% and a level of significance of 5%, at least 206 datasets would be needed. Since a dropout rate of about 5-10% was estimated, the plan was to recruit a total of 220 subjects. Complete case analyses were performed. Missing values from completers concerning secondary outcomes were substituted by the ‘last-observation-carried-forward’ principle. The Mann–Whitney test and linear regression analysis with adjustment for baseline Hba1c, age, sex, and weight difference was used to analyze differences between groups, Bonferroni's Multiple Comparison Test for analyses of repeated measurements within groups and the Wilcoxon signed rank test to determine if differences deviated from 0. Fisher’s exact test was used to analyse differences between groups or time points. The level of significance was 0.05. The Bonferroni correction was used for multiple testing (n = 15) resulting in p = 0.05/15 = 0.033. Statistical analyses were performed using GraphPad Prism 4.03 (GraphPad Software, San Diego, CA, USA) and SAS statistical package version 9.1 (SAS Institute, Cary, NC, USA).

## Results

### Sample characteristics

**Figure 1:**
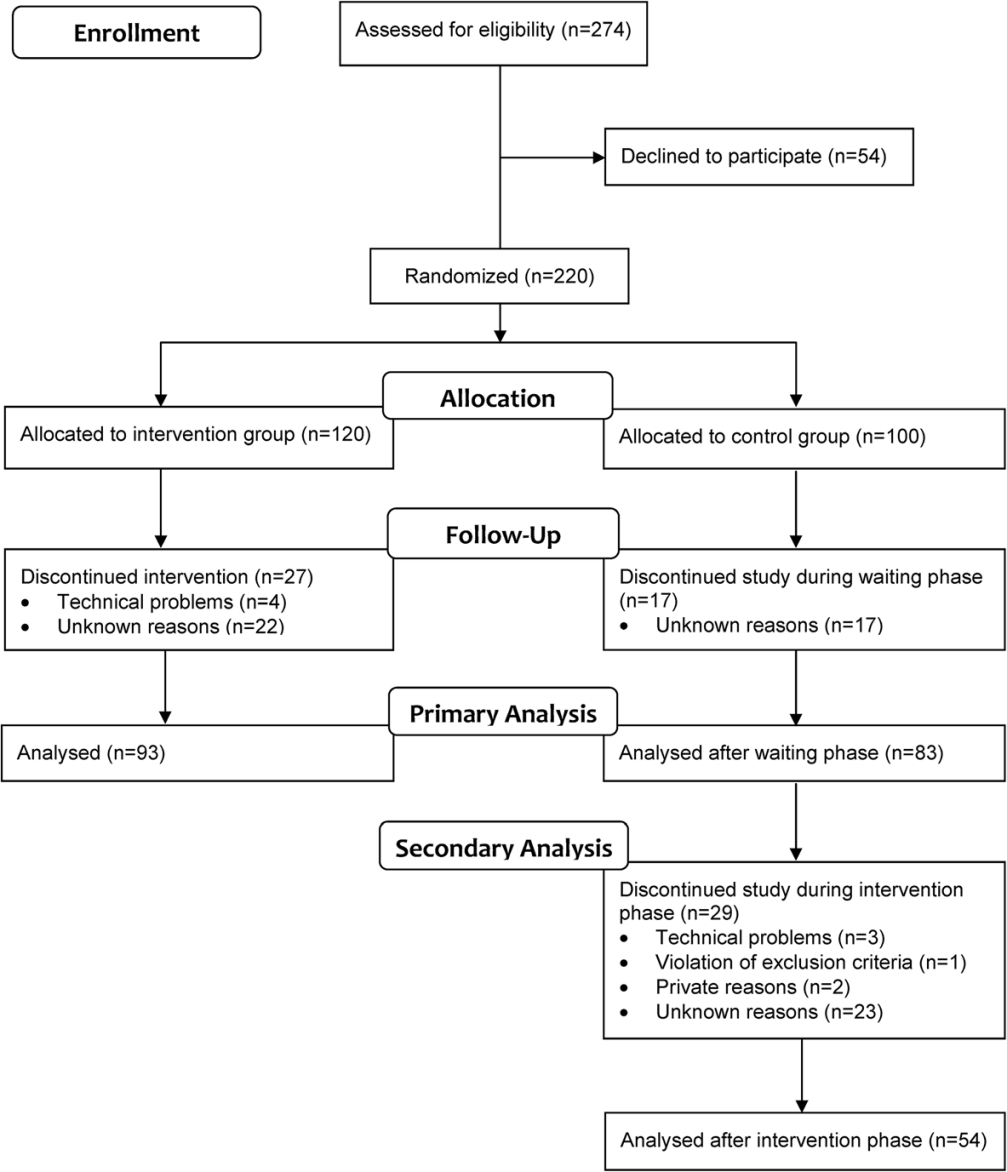
Flowchart of study participants.

220 T2DM patients were randomly assigned to the intervention (n = 120) and the control group (n = 100; Figure 1). Baseline parameters were not significantly different (Table 1). 93 (77.5%) participants of the intervention group completed the study. In the control group 83 (83.0%) finished the waiting phase and 54 (54.0%) the 12-week intervention. The dropout rate did not differ significantly between both groups. The drop outs did not significantly differ from completers concerning their baseline characteristics (Table 2).

**Table 1:** Baseline characteristics and study effects

**Parameters**	**Intervention group (n = 120)**				**Control group (n = 100)**			
**Sex (male/female) [n]**	**55 (46%) / 65 (54%)**				**46 (46%) / 54 (54%)**			
**Age [years]**	**62 ± 11**				**60 ± 9**			
	**Baseline (n = 120)**	**End (n = 93)**	**∆**	**p**	**Baseline (n = 100)**	**End (n = 83)**	**∆**	**p**
Weight [kg]	98 ± 19	96 ± 19	-1.2 ± 4.7	0.0001	96 ± 22	96 ± 24	-0.7 ± 2.6	0.014
Body mass index [kg/m^2^]	34.1 ± 6.5	33.5 ± 6.5	-0.4 ± 1.6	0.0001	33.2 ± 6.3	33.2 ± 6.5	-0.3 ± 0.9	0.007
Systolic blood pressure [mmHg]	134 ± 15	132 ± 13	-2 ± 15	ns	135 ± 17	136 ± 17	2 ± 14	ns
Diastolic blood pressure [mmHg]	80 ± 8	79 ± 8	-0.6 ± 10	ns	80 ± 9	80 ± 8	-0,3 ± 9	ns
Fasting blood glucose [mg/dl]	136 ± 39	127 ± 37	-8 ± 35	0.041	130 ± 39	130 ± 37	-3 ± 24	ns
Total cholesterol [mg/dl]	207 ± 43	204 ± 44	-3 ± 23	ns	202 ± 45	201 ± 45	-2 ± 18	ns
HDL cholesterol [mg/dl]	50 ± 14	51 ± 14	0 ± 7	ns	48 ± 11	49 ± 12	0 ± 5	ns
LDL cholesterol [mg/dl]	125 ± 35	125 ± 36	0 ± 23	ns	124 ± 50	120 ± 42	4 ± 26	ns
Triglycerides [mg/dl]	200 ± 123	198 ± 114	2 ± 65	ns	234 ± 251	238 ± 27	5 ± 69	ns
Physical activity [%]	42 ± 19	59 ± 17	12 ± 12	<0.0001	43 ± 21	46 ± 19	4 ± 10	0.002
Treatment with Metformin [%]	73	72	-1	ns	78	81	3	ns
Treatment with DPP-4 inhibitors [%]	16	16	0	ns	19	19	0	ns

HDL, high-density lipoprotein; LDL, low-density lipoprotein. Shown are means ± standard deviations. Self-reported physical activity was estimated according to a 6-point questionnaire asking for intensity of working time physical activity, intensity of leisure time physical activity, frequency of physical activity during winter time, frequency of physical activity during summer time, duration of walking time per day and duration of bicycling time per day. Single values were summed up with the maximal reachable value set to 100%. Differences between groups had been determined by Chi-square test and Mann Whitney test.

**Table 2:** Baseline characteristics of completers and dropouts

**Parameters**	**Intervention group (n = 120)**		**Control group (n = 100)**	
	**Completers (n = 93)**	**Dropouts (n = 27)**	**Completers (n = 83)**	**Dropouts (n = 17)**
Sex (male/female) [n]	42 / 51 (45% / 55%)	13 / 14 (48% / 52%)	35 / 48 (42% / 58%)	11 / 6 (65% / 35%)
Age [years]	61 ± 7	61 ± 9	60 ± 8	63 ± 7
Weight [kg]	97 ± 19	98 ± 21	97 ± 23	92 ± 13
Body mass index [kg/m^2^]	33.9 ± 6.4	34.4 ± 7.1	33.5 ± 6.5	31.7 ± 4.6
Systolic blood pressure [mmHg]	134 ± 15	135 ± 13	134 ± 17	138 ± 17
Diastolic blood pressure [mmHg]	79 ± 8	81 ± 9	80 ± 10	80 ± 5
Fasting blood glucose [mg/dl]	134 ± 38	141 ± 41	133 ± 40	118 ± 26
Total cholesterol [mg/dl]	207 ± 42	207 ± 45	203 ± 47	198 ± 38
HDL cholesterol [mg/dl]	50 ± 14	49 ± 8	48 ± 11	46 ± 9
LDL cholesterol [mg/dl]	125 ± 34	128 ± 31	124 ± 48	123 ± 37
Triglycerides [mg/dl]	209 ± 128	179 ± 84	243 ± 257	188 ± 70
Physical activity [%]	42 ± 18	40 ± 23	41 ± 19	52 ± 28
Treatment with Metformin [%]	77	59	78	76
Treatment with DPP-4 inhibitors [%]	14	22	19	18

For details please see legend to Table 1.

### Changes in HbA1c

**Figure 2:**
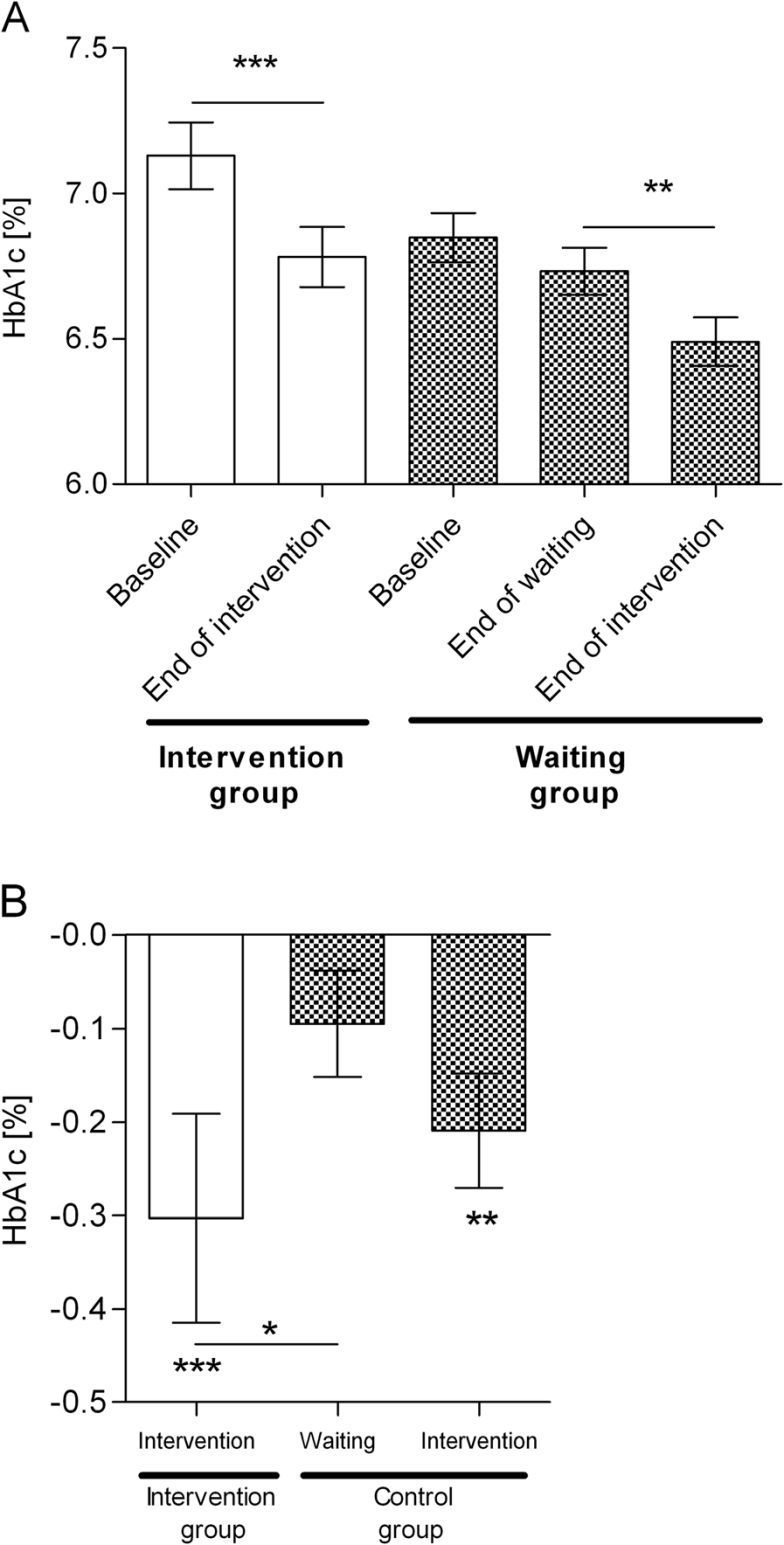
**Significant reduction of HbA1c during the 12-week exercise game intervention.** Shown are means ± standard error of means. **(A)** HbA1c values have been determined at baseline and after 12 weeks in the intervention group (n = 93; white bars) and the control group (n = 83; checked bars) as well as after additional 12 weeks of intervention (n = 54). **(B)** Differences in HbA1c were defined as values after 12 weeks of intervention or waiting minus baseline values, and additionally for the control group, values after 12 weeks of intervention minus values after 12 weeks of waiting. Differences between groups have been estimated using the Mann–Whitney test and within groups using the Wilcoxon signed rank test; Wilcoxon signed rank test was used to determine if differences were significantly different from 0 (*, p < 0.05; **, p < 0.01; ***, p < 0.001).

The complete case analysis demonstrated that, during the 12 weeks of intervention, patients in the intervention group (n = 93) were able to significantly reduce their HbA1c from 7.1 ± 1.3% to 6.8 ± 1.0%; p = 0.0002; Figure 2A) while no significant reduction (from 6.8 ± 0.9% to 6.7 ± 0.7%) was seen in the control group (n = 83) during the waiting phase. In detail, the reduction in HbA1c differed significantly from zero in the intervention group only (-0.3 ± 1.1%; Figure 2B), but not in the control group (-0.1 ± 0.5%). HbA1c values were not significantly different between groups at baseline or after the 12 week study period. Nevertheless, the within group differences reached statistical significance (p = 0.01), and these differences remained statistically significant after adjustment for baseline HbA1c and further potential confounders (Table 3). Even after adjustment for weight reduction, which might be considered as a mediator for HbA1c improvement, the effect remained significant. The percentage of participants who reached the treatment goal of HbA1c <7.0% increased in the intervention group from 56 to 65% (+9%) and decreased in the control group from 68 to 67% (-1%). When the control group finished the waiting phase and also started intervention, a significant reduction of -0.2 ± 0.5% of HbA1c (p = 0.003) was observed in a secondary analysis, leading to 78% reaching the treatment goal. When an intention-to-treat analysis was performed results stayed the same with a significant reduction of HbA1c of -0.24 ± 0.96% (from 7.1 ± 1.3% to 6.9 ± 1.1%; p = 0.0002) in the intervention group vs. -0.08 ± 0.47% in the control group (from 6.8 ± 0.9% to 6.8 ± 0.8%). For the type of intervention (exercise gaming vs. waiting) an independent impact (p = 0.005) on HbA1c reduction was observed even after adjustment for baseline HbA1c and further potential confounders (data not shown). The results even remain robust after Bonferroni correction for multiple testing.

**Table 3:** Effect on HbA1c reduction

	**Unadjusted model**		**Adjusted model 1**		**Adjusted model 2**		**Adjusted model 3**	
	**Effect size (SEM)**	**p**	**Effect size (SEM)**	**p**	**Effect size (SEM)**	**p**	**Effect size (SEM)**	**p**
**Intervention**	-0.332 (0.110)	0.010	-0.229 (0.091)	0.012	-0.228 (0.092)	0.014	-0.211 (0.089)	0.018
**Baseline HbA1c**	-	-	-0.396 (0.043)	<0.0001	-0.397 (0.046)	<0.0001	-0.384 (0.044)	<0.0001
**Age**	-	-	-	-	-0.001 (0.001)	0.885	-0.003 (0.006)	0.665
**Sex**	-	-	-	-	0.025 (0.092)	0.790	0.009 (0.089)	0.924
**Weight difference**	-	-	-	-	-	-	0.042 (0.011)	0.0003

n = 93 + 83 = 176; Model 1: adjusted to baseline HbA1c; Model 2: adjusted to baseline HbA1c, age and sex; Model 3: adjusted to baseline HbA1c, age, sex and weight difference.

### Changes in cardiometabolic risk factors and physical activity

Significant reduction of weight and BMI was observed in both groups (Table 1). In the intervention group FBG levels also improved significantly although the reduction no longer remained statistically significant after correction for multiple testing. Patients in the intervention group reported an increase in physical activity beyond their activity using the exercise game in everyday life (i.e. gymnastics, walking, bicycling). Their physical activity increased more significantly during the 12 weeks of intervention than those of participants in the control group, although they were also more physically active during the waiting phase. When the control group started the exercise game intervention they also experienced weight reduction by -1.8 ± 3.1 kg of (p < 0.001), BMI by -0.6 ± 1.0 kg/m^2^ (p < 0.001), FBG by -7.0 ± 20.4 mg/dl (p = 0.008), and further increased their physical activity by 10 ± 10% (p < 0.001). Most of participants did not make use of the exercise game alone but played together with family members (61% in the intervention and 63% in the control group, i.e. partners (40% vs. 41%), children (30% vs. 11%), grandchildren (23% vs. 8%) and other family members (6% vs. 1%)). Medication levels with Metformin and DPP-4 inhibitors did not change significantly and no adverse or side effects were reported.

### Changes in diabetes-dependent impairment, subjective wellbeing and quality of life

**Figure 3:**
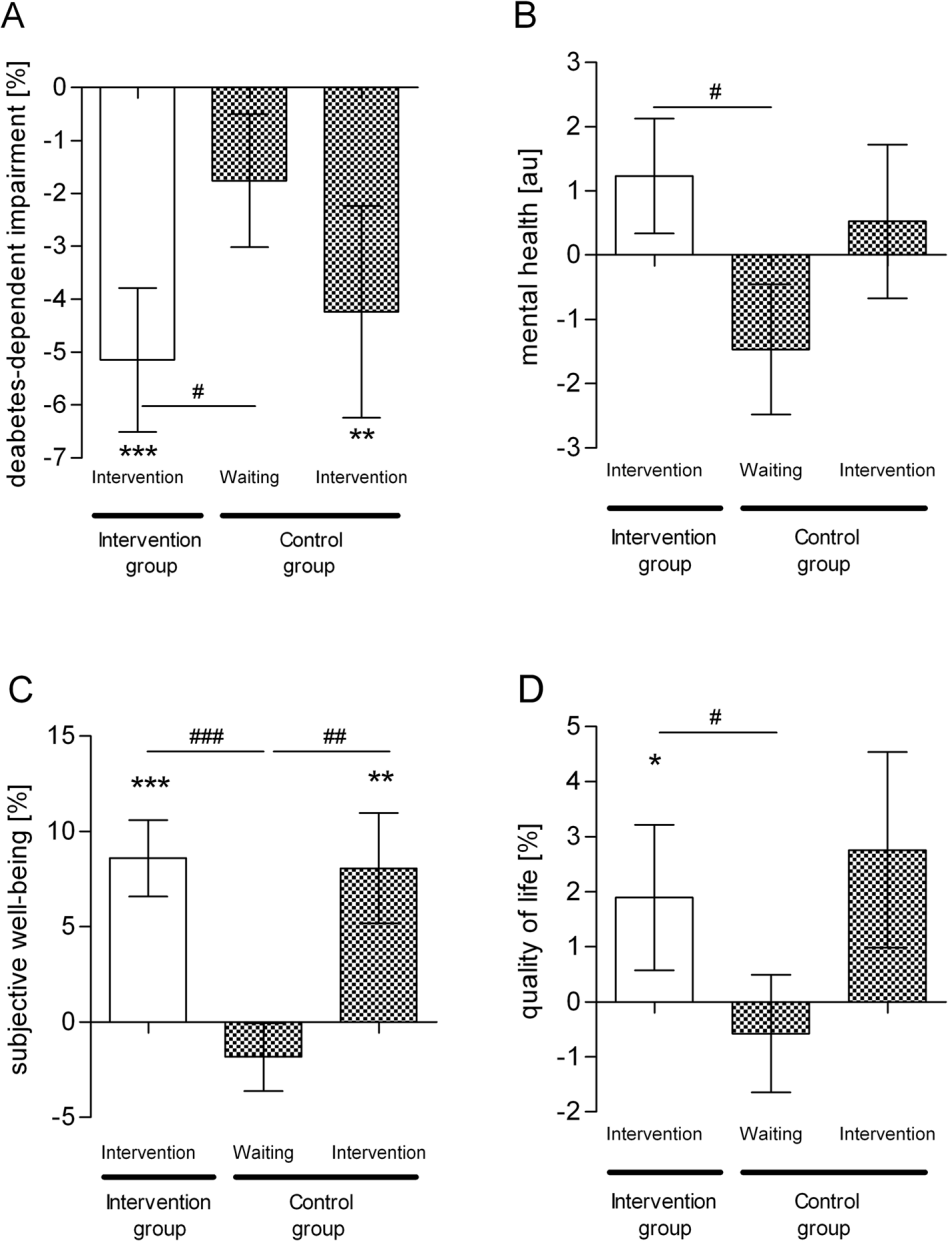
**Improvement in diabetes-dependent impairment, subjective wellbeing and quality of life during exercise game intervention.** Using validated questionnaires we estimated **(A)** diabetes-dependent impairment (PAID), **(B)** mental health (SF-12), **(C)** subjective wellbeing (WHO-5) and **(D)** quality of life (ADS-L). For details see legend to Figure 1.

Diabetes-dependent impairment was significantly reduced in the intervention group compared to the control group (p = 0.03). In detail, there was a significant reduction of diabetes-dependent impairment in both groups during intervention phases, with a reduction of 5.2 ± 13.2% in the intervention group (p < 0.001) and 4.2 ± 14.7% in the control group (p = 0.008), while reduction was not statistically significant during the waiting phase (Figure 3). In addition, while mental health improved in the intervention group, it deteriorated in the control group during the 12-week study (p = 0.02); changes in physical health were not significantly different (data not shown). Subjective wellbeing significantly improved in the intervention group compared to the control group (p < 0.001). Wellbeing increased significantly during the 12-week intervention by 8.6 ± 19.4% (p < 0.001) in the intervention group and 8.1 ± 21.3% in the control group (p = 0.004), but did not change significantly during the waiting phase. Finally, quality of life increased significantly in the intervention group by 2.4 ± 12.9% (p = 0.03) compared to the control group, which showed a slight decrease during waiting phase (p = 0.05).

### Changes in depression

**Figure 4:**
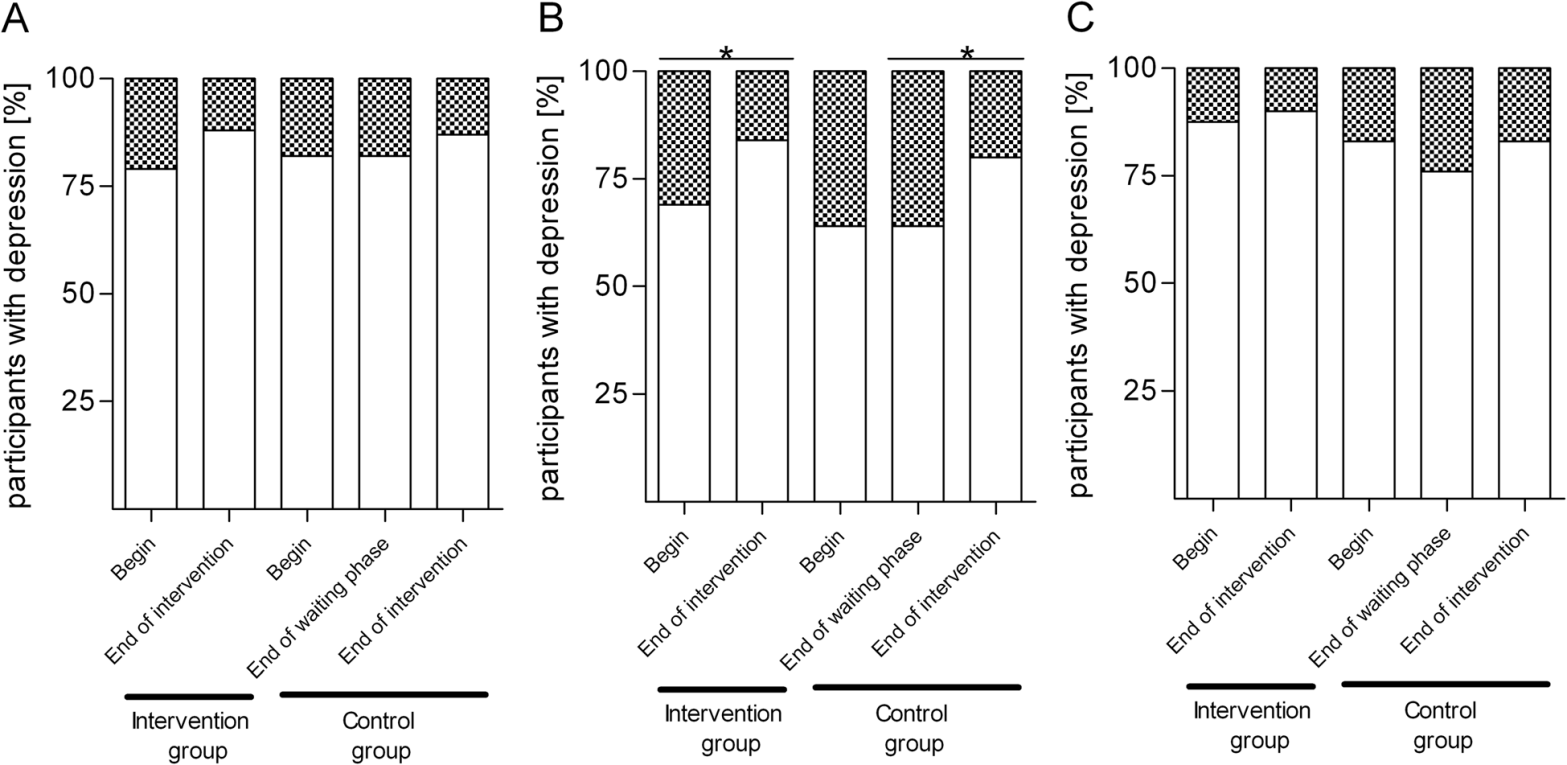
**Percentage of patients with depression.** Depression was self-assessed using the validated questionnaires **(A)** PAID, **(B)** WHO-5 and **(C)** ADS-L. Participants who reached a score >39% (PAID), ≤52% (WHO-5) and ≥40 (ADS-L) were diagnosed as depressed. The percentage of patients with depression is given by the checked part of the bar, the white part of the bar gives the percentage of participants without depression. Fisher’s exact test was used to analyze if the numbers of depressed participants differed between groups or points in time (*, p < 0.05).

Depression was measured using the validated questionnaires PAID, WHO-5 and ADS-L. With all instruments, the percentage of depressed patients had been comparable in both groups at baseline (PAID: 25 (21%) in the intervention vs. 18 (18%) in the control group; WHO-5: 37 (31%) vs. 36 (36%); ADS-L: 15 (13%) vs. 17 (17%)), although the percentage of patients diagnosed with depression differed according to the questionnaire used (Figure 4). All three measurements indicate that the percentage of depressed patients went down in both groups during exercise game intervention, although only depression measurement using the WHO-5 questionnaire reached a statistical significance (15 (16%) in the intervention group after intervention (p = 0.019); 30 (36%) vs. 11 (20%) in the control group comparing the waiting and the intervention phase (p = 0.018)). During the waiting phase the percentage of depressed patients remained stable.

## Discussion

To our knowledge this is the first randomized-controlled trial showing a benefit of exergaming for a clinical endpoint and for the quality of life of a disease. In this study we demonstrate a significant reduction of HbA1c, weight and BMI during the 12-week intervention. The majority of participants reported having played together with family members, and that the extent of their physical activity increased not only during exergaming but also in everyday life. Significant improvements in diabetes-dependent impairment, subjective wellbeing and quality of life as well as a reduction of depression were observed. Therefore, exercise games may potentially be used in a home setting as a tool to reduce sedentary behavior in T2DM.

It has been known for a long time that lifestyle intervention programs are successful for the treatment of patients with manifested T2DM. The Look-AHEAD (Action For Health in Diabetes) study [1,9] demonstrated that with a combination of a calorie restricted diet, exercise, motivation and self-monitoring of blood glucose, patients could achieve a decrease of mean HbA1c from 7.3 to 6.6%, an 8.6% weight loss, and a significant reduction of anti-diabetic medication within one year. After four years, HbA1c reduction was maintained at -0.36% and weight loss at -6.2% [9]. Also a recent meta-analysis of community-based physical activity for adults with type 2 diabetes revealed a significant lowering of HbA1c levels by -0.32% [95% CI -0.65, 0.01] [10]. However, it needs to be emphasized that such interventions required an enormous amount of personal and economical effort. In contrast, in our approach, mentoring and costs were low, just mailing the *Wii* console with balance board, and the exercise game *Wii Fit Plus*. For the point of view of the participants, the use of the game was a low-threshold proposal for lifestyle intervention. In detail, we did not test the effect of physical activity on glucometabolic control, but just the effect of providing a device for interactive exercise in order to encourage self-motivation in the participants. Nevertheless, a comparable HbA1c reduction of 0.3% was reached and the percentage of participants reaching the HbA1c goal of <7.0% [11] increased by 9%. A meta-analysis investigating the efficacy of pharmacological therapies demonstrated that the amount of HbA1c reduction essentially depends on baseline HbA1c [12]. HbA1c reduction seems to be greater the higher the baseline levels had been previously. With a mean baseline HbA1c of 7.0-7.9%, therapies with oral antidiabetic medication achieved an HbA1c reduction of 0.1%, while in our study the baseline HbA1c in the intervention group was 7.1 ± 1.3%, and a reduction of 0.3% was achieved.

Data about the use of exercise games for lifestyle intervention in the elderly are scarce, although a systematic review revealed significant health benefits in older adults [13]. For *Wii Fit*, especially, it has already been shown that regular use might be a vehicle for increasing physical activity in the elderly [14]. Moreover, cycling games seem to increase cognition in older adults [15]. It has also been used for lifestyle intervention in adolescents to encourage them to lose weight [16]. Overweight school children were encouraged to play an exercise game for 30-60 min/school day for a period of 20 weeks during lunch-time or after school. Adolescents played with or against a peer to expend calories, while in the control group participants played alone. Within this controlled setting, the highest weight loss with a mean of 1.7 ± 4.5 kg was achieved with cooperative playing, and also measurements for self-efficacy increased. This finding is fully in line with our results, demonstrating that the majority of participants preferred to play with family members rather than by themselves. Since key motivators to physical activity were weight management, feelings of physical and mental well being, as well as social relationships associated with physical activity, exercise games should help to motivate players to exercise, and could take advantage of group dynamics to motivate players in terms of the duration of the exercise period [17]. Obviously, the motivating effects are not only short-term but are maintained and sufficient to significantly reduce long-term blood-glucose parameters such as HbA1c. Another study including adolescents playing exercise games alone or with a virtually present partner demonstrated significantly higher persistence in all experimental conditions with the virtual partner present [18]. One explanation for this might be the finding that cooperative exercise gaming produces a higher intrinsic motivation that comes from a desire for control and this is related to a higher expenditure of energy [19]. Therefore, exercise games are mostly played in a social context [20]. This might not be true only for youngsters, but also for the elderly, since a meta-analysis reported significant mental health outcomes in the majority of the reviewed studies, resulting in positive consequences for physical and lastly social health in older adults [13].

Key motivations for playing exercise games were perceptions of enjoyment, feeling better afterwards and participation in a social context [21]. This is fully in line with our results, which demonstrated that during the 12-week exercise game intervention, diabetes-dependent impairment decreased, and mental health, subjective wellbeing and quality of life improved significantly. The combination of physical activity per se, the feeling of having done something positive for one’s health, and playing fun games with family members seems to strengthen patients’ motivation, resulting not only in weight loss and improvement of glucometabolic control but also in an positive attitude towards life.

Our study has several limitations that need to be considered. First, a completer rate of only 67% was achieved and of those a completer analysis had been performed. The patients who managed to improve their glucometabolic control may have been more strongly motivated to stay with the program. This might have biased the results and the effects might have been weakened in the complete study population. Nevertheless, the percentage of drop out in our study are comparable to those seen in other exercise interventions for older adults [22] and the fact that the baseline characteristics of completers and dropouts, the results of the intention-to-treat analysis as well as the outcomes in both groups after exercise intervention did not differ, argues against such a responder bias. Second, it might be seen as limitation of our study that the exercise game was used at home in an uncontrolled setting and that no objective information was obtained about the duration and intensity of exercising. However, it was our particular aim to create a situation close to real-life, where T2DM patients were not enclosed in an expensive and all-embracing mentoring program but had the opportunity of performing physical activities on their own in order to demonstrate the potential of a low-threshold intervention for clinical outcomes. Therefore, we refrained from close supervision. Third, data was self-reported and clinical parameters were not measured in a standardized manner but at local laboratories. This did not affect the intra-individual analysis because glucometabolic measurements were performed at baseline and at end of study in the same local laboratory and were reported in written form by the attending physician. In assessing physical activity, diabetes-dependent impairment, subjective wellbeing and quality of life, validated self-assessment questionnaires were used and the comparison baseline vs. end of study was performed using the same algorithm. It might be speculated that the patients participating in this study might have been more strongly motivated than the general T2DM population. Nevertheless, that might be true for all patients participating in clinical studies. Perhaps those patients who had heard about the Wii and exercise games from their children or grandchildren might have been more willing to participate, but generally, for all T2DM patients who are physically able, exercise games might offer an alternative form of home exercise.

## Conclusions

Our data demonstrated a significant improvement in glucometabolic control and quality of life during a low-threshold intervention with the interactive exercise game *Wii Fit Plus*. Given the positive attitudes of the participants and the limited restrictions for gaming at home, exercise games may potentially be used in a home setting as a tool to reduce sedentary behavior in T2DM. In future, exercise games should be specially developed for this group of patients, glucometabolic control should be optimized and, in order to increase adherence, online transfer of performed exercises, as well as telediabetological coaching, should be included in a treatment program.

## Competing interests

The study was funded by Novartis Pharma GmbH. The funding organization had no influence on design and conduct of the study; collection, management, analysis, and interpretation of the data; and preparation, review, or approval of the manuscript. KK and SM received a research grant from Novartis Pharma GmbH. The authors were not remunerated to write this article. Wii consoles, balance boards and the exercise games Wii Fit Plus were provided by Nintendo of Europe GmbH.

## Authors’ contributions

KK and SM are responsible for conception and design, analysis and interpretation of data. KK drafted the article; SM revised it critically for important intellectual content. KK and SM gave final approval of the version to be published. KK and SM are guarantors of the paper. KK and SM accept full responsibility for the work and/or the conduct of the study, had access to the data, and controlled the decision to publish. Both authors read and approved the final manuscript.

## Pre-publication history

The pre-publication history for this paper can be accessed here:

http://www.biomedcentral.com/1472-6823/13/57/prepub
